# Internet-based monitoring of influenza-like illness (ILI) in the general population of the Netherlands during the 2003–2004 influenza season

**DOI:** 10.1186/1471-2458-6-242

**Published:** 2006-10-04

**Authors:** Richard L Marquet, Aad IM Bartelds, Sander P van Noort, Carl E Koppeschaar, John Paget, François G Schellevis, Jouke van der Zee

**Affiliations:** 1 NIVEL (Netherlands Institute for Health Services Research), the Netherlands, P.O. Box 1568, 3500BN, Utrecht, The Netherlands; 2 Instituto Gulbenkian de Ciência, Apartado 14, 2781-901 Oeiras, Portugal; 3 The Great Influenza Survey, Burgemeester Boreelstraat 13, 2013 BT Haarlem, The Netherlands

## Abstract

**Background:**

An internet-based survey of influenza-like illness (ILI) – the Great Influenza Survey or GIS – was launched in the Netherlands in the 2003–2004 influenza season. The aim of the present study was to validate the representativeness of the GIS population and to compare the GIS data with the official ILI data obtained by Dutch GPs participating in the Dutch Sentinel Practice Network.

**Method:**

Direct mailings to schools and universities, and repeated interviews on television and radio, and in newspapers were used to kindle the enthusiasm of a broad section of the public for GIS. Strict symptomatic criteria for ILI were formulated with the assistance of expert institutes and only participants who responded at least five times to weekly e-mails asking them about possible ILI symptoms were included in the survey. Validation of GIS was done at different levels: 1) some key demographic (age distribution) and public health statistics (prevalence of asthma and diabetes, and influenza vaccination rates) for the Dutch population were compared with corresponding figures calculated from GIS; 2) the ILI rates in GIS were compared with the ILI consultation rates reported by GPs participating in the Dutch Sentinel Practice Network.

**Results:**

13,300 persons (53% of total responders), replied at least five times to weekly e-mails and were included in the survey. As expected, there was a marked under-representation of the age groups 0–10 years and 81->90 years in the GIS population, although the similarities were remarkable for most other age groups, albeit that the age groups between 21 and 70 years were slightly overrepresented. There were striking similarities between GIS and the Dutch population with regard to the prevalence of asthma (6.4% vs. 6.9%) and the influenza vaccination rates, and to a lesser degree for diabetes (2.4% vs. 3.5%). The vaccination rates in patients with asthma or diabetes, and persons older than 65 years were 68%, 85%, and 85% respectively in GIS, while the corresponding percentages in the Dutch population were 73%, 85% and 87%. There was also a marked similarity between the seasonal course of ILI measured by GIS and the GPs. Although the ILI rate in GIS was about 10 times higher, the curves followed an almost similar pattern, with peak incidences occurring in the same week.

**Conclusion:**

The current study demonstrates that recruitment of a high number of persons willing to participate in on-line health surveillance is feasible. The information gathered proved to be reliable, as it paralleled the information obtained via an undisputed route. We believe that the interactive nature of GIS and the appealing subject were keys to its success.

## Background

The internet has changed the way we live and work, and may also become an important tool for epidemiological studies and health surveillance. To achieve this goal, it is mandatory to first assess the accuracy and reliability of communicating health information via internet. This may be especially relevant when there is public anxiety due to bio-terrorist threats or anticipated outbreaks of pandemic infectious diseases [1,2].

Internet-based surveys may be conducted by means of interactive interviews or by questionnaires designed for self-completion. A major concern of web-based surveys is the non-representative nature of the internet population and the self-selection of participants and it is generally assumed that the results are likely to be biased; firstly because internet has not penetrated equally into all age-groups, and secondly because people who do respond almost certainly have different characteristics from those who do not. It has been argued, however, that internet samples may actually be more representative than traditional samples [3]. When traditional paper-and-pencil methods were compared with internet samples, it was observed that internet samples were more diverse with respect to gender, socioeconomic status, geographic region and age. Moreover, internet surveys were less affected by non-serious responders and were found to be highly consistent with findings from traditional methods [4].

Influenza is an important public health problem in the industrialized world. It is associated with high general practice consultation rates, increased hospital admissions and excess deaths, and national networks for clinical and virological surveillance of influenza have existed in Europe since the 1950s. The first European influenza surveillance project was the Eurosentinel scheme, which was followed in 1992 by the ENS-CARE Influenza Early Warning Scheme and in 1996 by the European Influenza Surveillance Scheme (EISS); a total of 21 EU and 3 non-EU states are currently members of EISS [5]. The clinical surveillance of influenza by EISS is predominantly based on reports made by general practitioners (GPs) participating in a national or regional sentinel surveillance system. They usually represent 1–5% of the GPs working in the country or region. In the Netherlands, the Dutch Sentinel Practice Network consists of a representative sample of about 60 GPs covering about 1% of the Dutch population, and is the national provider of data for EISS [6,7]. In winter, the sentinel GPs are asked to take nose and/or throat swabs from patients with ILI for virological determination and these data function as the official data on which national and international decisions by health policy-makers are based.

An internet-based Great Influenza Survey (GIS) was launched in the Netherlands and the Dutch-speaking part of Belgium (Flanders) in 2003 [8]. The initiative for GIS was taken by one of us (CEK), who is editor in chief of a government-sponsored Dutch Internet Knowledge Portal (Kennislink) [9]. The objective of the portal is to make scientific information accessible to a broad public, and to kindle students' enthusiasm for science. Being a subject with a high general appeal, ILI was chosen as a vehicle to promote participation in an interactive enterprise in which the participant could experience the sensation of being a genuine scientist.

The aim of the present study was to validate and compare the internet-based GIS data with the official ILI data obtained by Dutch GPs participating in the Dutch Sentinel Practice Network during the 2003–2004 influenza season.

## Methods

### Design of GIS

In September 2003 the Dutch Knowledge Portal started its canvassing campaign to encourage a broad section of the public to participate in GIS. A task force that included experts on virology, public health, mathematics, science writing and informatics had been set up earlier that year to give the project a solid basis. The educational and scientific aims of the project were explained in direct mailings to schools and universities, and in repeated interviews on television and radio and in newspapers. Schools were provided with educational material on influenza to promote incorporation of GIS in lessons on biology and science, while a wealth of information on influenza, ILI and the objectives of GIS, written in appealing and accessible language, was provided on the GIS website that was launched in September 2003. Visitors to the website were invited to become GIS participants by providing personal information on gender and age, and by answering questions on health and lifestyle as summarized in table 1.

Participants received a weekly e-mail in which they were asked to answer questions about ILI symptoms they might have experienced since their last survey. (table 2). Strict symptomatic criteria were formulated in collaboration with scientists from the Netherlands Institute for Health Services Research (NIVEL) and the National Institute for Public Health and the Environment (RIVM), in order to distinguish ILI from a common cold. The criteria for ILI were: 1) acute onset (a prodromal stage of no more than 4 days); 2) sudden fever, (body temperature of at least 38°C), accompanied by; 3) at least one of the following respiratory symptoms: cough, running nose, sore throat and chest pain, plus; 4) headache or muscle pain. Only participants who responded at least five times to requests for information about their health were included in the analysis, in order to exclude unreliable information from incidental, non-serious visitors to the website. Data from first-time visitors with or without symptoms of ILI, who frequently joined GIS only when ILI was already epidemic, were also excluded to eliminate incorrect peaks in the incidence of ILI. The daily incidence of ILI was calculated on the basis of the responses of the active participants. The day on which the fever started was taken as the day of the onset of ILI. The different steps in the procedure, running from: 1) exclusion or inclusion of an ILI-patient on the basis of symptoms and frequency of reporting, 2) determination of the onset of symptoms by interpolation from previous and current responses, and 3) calculation of daily incidence were executed via a computer program specifically written for GIS. Implementation of these steps, by which real-time GIS data were converted to more reliable daily ILI rates, took 4 days. Data were collected from week 44/2003 until week 14/2004. The GIS website continuously provided up to date feed-back information on how the situation in the Netherlands was evolving during the influenza season, and related heath information, written in a comprehensible style, was provided. If differences were noted between the course of ILI based on GIS and the sentinel GPs, this was discussed on the website to keep the participants involved and motivated. The Dutch speaking part of Belgium (Flanders) also participated in GIS, but the data from Belgium are not included in the current analysis.

### Did the GIS population reflect the Dutch population?

To address this question, some key demographic and public health statistics for the Dutch population were compared with corresponding figures calculated from GIS.

#### Demographics

The demographic data of the Dutch population provided by Statistics Netherlands as of 1 January 2004 [10] were compared with the demographic data of the GIS participants who responded frequently.

#### Prevalence of asthma and diabetes and influenza vaccination rates

Data on the prevalence of asthma and diabetes, obtained from the National Information Network of GPs (LINH), were compared with the prevalence of these (self- reported) conditions in the GIS population. LINH is a GP sentinel network consisting of about 80 GPs, equally distributed over the Netherlands and representative of the Dutch population [11]. The network provides encoded patient information extracted from electronic medical records, and data on the prevalence of asthma and diabetes reported by LINH for 2003 [12,13] were used for the present study. Vaccination uptake rates were also obtained from LINH, since Dutch GPs have been offering influenza vaccination to all their high-risk patients since 1996, in accordance with the guidelines of the Dutch College of General Practitioners. In the present study, the vaccination rates of patients with asthma or diabetes, and persons older than 65 years as supplied by LINH in 2003, were compared with the rates of (self-reported) vaccination of these groups in GIS [14].

### ILI rates during the 2003–2004 influenza season, comparison between GIS and the Dutch Sentinel Practice Network

Fifty-six GPs participated in the Dutch Sentinel Practice Network in 2003–2004. As they have been doing since 1970, participating GPs reported new cases of ILI each week, using specific forms that were sent to our Institute by mail or fax. The number of ILI consultations per week was taken as the numerator and the total number of patients in the participating sentinel practices (1% of the Dutch population) as the denominator to calculate the consultation rates for ILI. The Dutch data are placed weekly on the EISS website, together with the data from other countries joined together in EISS. The published weekly consultation rates (incidence)/100,000 occurring in the 2003–2004 influenza season [5] were used for the present study. The daily incidences of ILI calculated by the computer for GIS were converted into weekly incidences to enable the comparison between EISS and GIS.

### Ethical approval

The study was carried out according to Dutch legislation on privacy. The privacy regulation of the study was approved by the Dutch Data Protection Authority. According to Dutch legislation, obtaining informed consent is not obligatory for observational studies.

## Results

### Comparison of demographic data

**Figure 1:**
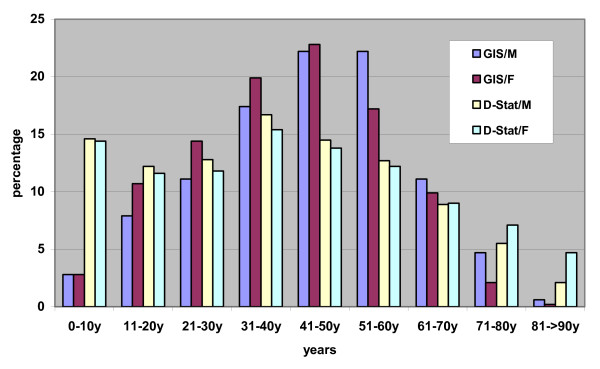
Age distribution of the Male (M) and Female (F) GIS population and the Dutch (D-Stat) population.

13,300 persons (53% of total responders) replied at least 5 times to the weekly e-mails sent by GIS to inquire about ILI. This sample of dedicated responders was used in the current analysis. According to Statistics Netherlands, the male-female ratio of the Dutch population in 2004 was 49/51% [10] and there was a slight overrepresentation of women in the GIS population, where the male-female ratio was 47/53%. The age distributions of the Dutch population (as provided by Dutch Statistics) and the GIS population are presented in figure 1. As could have been expected, there was a significant underrepresentation of the age groups 0–10 years and 81->90 years in the GIS population (see odds ratios table 3). In addition, there was a moderate underrepresentation of predominantly males in the age group 11–20 years and of females in the age group 71–80 years. The age groups between 21 and 70 years were all overrepresented in GIS. It is interesting to note that this was mainly due to excess in female responders up to the age of 50 years, thereafter by excess in male responders.

### Comparison of the prevalence of asthma and diabetes, and influenza vaccination rates in GIS and LINH population

The results are summarized in table 4. The appreciable similarities between the data provided by GIS and LINH are remarkable. The prevalence of asthma calculated for GIS was 0.5% higher than measured by LINH (6.9% vs. 6.4%), whereas the prevalence of diabetes was 1.1% lower (2.4% vs. 3.5%). This latter difference may be due to the lower proportion of elderly persons in the GIS population. Although the vaccination rates in the GIS risk-populations were somewhat lower than in the LINH population, the similarities outnumbered the differences nevertheless, implying a high level of health equivalence between the Dutch and GIS populations.

### Comparison between ILI rates in GIS and the Dutch Sentinel Practice Network

**Figure 2:**
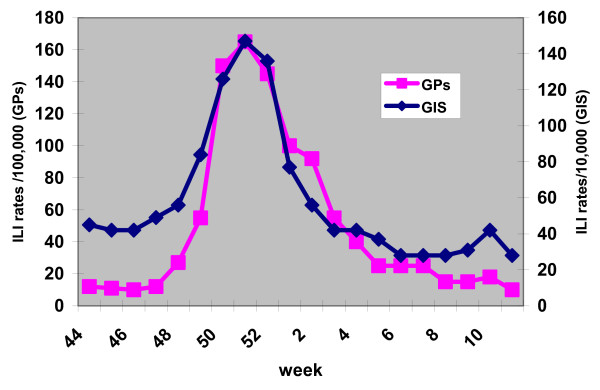
ILI rates measured by GPs and the internet-based Great Influenza Survey (GIS) during the 2003–2004 influenza season.

The incidence curves of ILI per 10,000 persons according to monitoring by GIS and per 100,000 for the Dutch Sentinel Practice Network are shown in figure 2. It is interesting to note that the two curves followed an almost identical course throughout the ILI season, with peak incidences occurring in the same week. (If the unabridged data of all GIS participants were take into account, thus including the short-time responders and those who started to participate when ILI was already epidemic, the GIS curve not only showed an early peak but remained at a higher level throughout the season; data not shown). The mean incidence of ILI was about ten times higher in the GIS population throughout the whole observation period. However, the baseline values in weeks 46–47 differed by a factor of 40–50 and in weeks 8–11 by a factor 20–30. According to criteria set by the National Influenza Centre, NIVEL and RIVM, ILI starts to become epidemic when the weekly incidence exceeds 30/100,000 [14]. This occurred in week 48. Figure 2 shows that incidence gained momentum as from week 48 and peaked in week 51. An almost similar course was observed for GIS; the incidence rate was already starting to increase in week 46, but the exponential rise also began in week 48. The incidence rates in GIS and EISS decreased in a comparable fashion after week 51 and had returned to background levels by week 8.

## Discussion

According to estimates made in 2003 by Statistics Netherlands, 6 out of 10 inhabitants of the Netherlands older than 12 years were regular internet users. Internet usage was high (75%–85%) between the age of 12–45 years, steadily decreasing thereafter by 10–15% per decade. About 5–8% of elderly persons over 75 years still used internet regularly. Internet usage by men was slightly higher than by women in 2003 and the higher the level of education, the higher the use [10]. Where the accrual of persons willing to participate in GIS was concerned, the enterprise was definitely a success. Following substantial recruitment efforts in the media, almost 26,000 persons replied to the requests for participation by answering the intake questions at least, and 53% of these became serious participants. This percentage compares favorably with the 51.2% of serious participants who finished a Swedish internet survey on sexual behavior following an open invitation via a website, and the 50%-plus response rate obtained in a population web survey on lifestyle issues reported by Augustsson et al. [16,17]. Much lower rates have been reported for online health surveys, however, possibly because they involved less appealing subjects (than sex or influenza), had a poorer design, or were less creative in their motivation and incentive strategy [18,19].

The serious participants in our study were not equally distributed with regard to gender and age. The Dutch population has an almost equal male-female distribution up to the 71–80 years age group. In the GIS population, however, women between 11 and 50 years were clearly overrepresented, whereas the reverse was observed after the age of 50 and men became overrepresented. Apart from gender, there was, by and large, a clear overrepresentation of GIS participants between 21 and 70 years and a marked under-representation before and after these age limits. The Swedish internet survey mentioned above found an almost identical overrepresentation of participants between 25 and 65 years and under-representation beyond these age limits [17]. A disproportionate distribution of this kind is not a great problem for many health-related issues, however, because there is a good chance that most relevant age groups are included. On the other hand, this imbalance may be a problem for the surveillance of topics such as ILI, because the very young, and the very old to a lesser extent, are largely underrepresented, while these are precisely the age groups that suffer most from ILI. We found, nevertheless, that this concern did not translate into a difference between the course of the ILI incidence curves from the Dutch Sentinel Practice Network and GIS. The incidence of ILI in the very young and old appears to have been less than had been expected, or was outnumbered by the magnitude of the ILI incidence in the rest of the GIS population. This was supported by the fact that the similarities between the 2 curves remained the same when very young and very old patients were excluded from the data provided by the GPs (manuscript in preparation). The major difference between the two curves is that the GIS curve started from a much higher background level and remained at a higher level throughout the season.

The most likely reasons for this difference are that only some patients with ILI complaints will go to their GPs, and that GPs are more critical in making the diagnosis. Part of the difference in amplitude between the GIS curve and the GP curve may also have been due to overrepresentation of GIS participants between 25 and 65 years of age. They represent the majority of the working population, and may be less likely to consult their GP for ILI complaints than the non-working population. On the other hand, they also represent the more healthy proportion of the population. It has been estimated earlier that in the Netherlands the incidence of ILI in the community at least is 6 times higher than presented to the GP [20], which corresponds with the present observation that the overall difference between GP consultation rate and GIS incidence is 10-fold.

It was a remarkable and encouraging observation that the course of ILI monitored by GIS so closely resembled the official survey of ILI by GPs participating in EISS. The implications of this finding for the surveillance of ILI itself are limited, however. The monitoring of ILI and influenza by EISS is well established and has not only proved to be reliable over the years, but also to function as an important source of scientific information with regard to virology and vaccination [15]. The key role of GPs in the EISS surveillance system ensures a high level of continuity and scrutiny, characteristics that are questionable in GIS. As far as continuity is concerned, GIS is being carried out for the third time in the Netherlands and Flanders in the 2005–2006 season and was started in Portugal for the first time [21]. It remains to be seen how successful these surveys will continue to be in the long run.

A comparable population-based approach for ILI surveillance, in which the GP is bypassed and information is provided by callers to a helpline, (NHS Direct), has been used in the UK. NHS Direct is a nurse-led telephone helpline providing health advice 24 hours a day. On receipt of a call and description of the caller's symptoms, nurses use their judgment to select the appropriate diagnostic algorithm in their computer, leading to self-care advice or a referral to another part of the NHS [22]. In principle, the call data, which can be analyzed daily or even by the hour, can also be used for community surveillance, including ILI. The usefulness of NHS Direct for ILI surveillance has been evaluated for two influenza seasons thus far and, although the results have been reassuring, the system was hindered by insufficient population coverage, lack of uniformity and poor definition of ILI [23,24].

There was some indication that the incidence of ILI started to increase 1–2 weeks earlier than reported by EISS, but this could not really be substantiated, due to the small number of early events and the high background level of ILI in GIS. For a better understanding of the natural course of ILI it might have been better if GIS had started much earlier than the start of the influenza season. It might have provided us with a badly needed threshold value.

It should be noted that in GIS it took about 4 days before reliable incidence rates could be deduced from real-time data, giving the internet-based approach a head start of about 5 days on the information provided by GPs. This difference between mail and web is likely to disappear as soon as Dutch GPs start reporting their data on a real-time basis. The success of an approach of this kind has been reported for GPs participating in a real-time influenza surveillance projects in other countries [25,26].

The major finding of the current study is that it is possible to recruit of a high number of persons, willing to participate in on-line health surveillance. This has been previously demonstrated for well-defined groups such as students [27], but only occasionally for the general population [16]. Our study demonstrates that not only was recruitment successful, but also that the information provided was reliable, because it paralleled the information that was gathered via a different, acknowledged route. In addition, the demographic and health characteristics of the participants were remarkably similar to the general Dutch population, apart from some typical deviations related to internet use. It can therefore be envisioned that this type of interactive on-line surveillance is extended to other health-related issues with, preferentially, a high public appeal such as obesity, life style and stress. Actually, questions about stress and stressful behavior have been asked to participants of the third GIS that took place during the 2005–2006 influenza season [8].

Bälter et al. recently made some wise remarks on the appropriate use of internet as a tool for epidemiological research [28], but their remarks were mainly devoted to technical subjects such as length and layout of the questionnaires. The GIS study allows us to tentatively point to another cornerstone that may be important for success: feedback of information to keep the participants involved and motivated.

## Conclusion

The current study demonstrates that recruitment of a high number of persons willing to participate in on-line health surveillance is feasible. The information gathered via internet proved to be reliable, because it paralleled the information obtained via an undisputed route. We believe that the interactive nature of GIS and the appealing subject were keys to its success.

## Competing interests

The author(s) declare that they have no competing interests.

## Authors' contributions

**RLM **performed the analysis of the data and drafted the manuscript.

**AIMB **participated in the design and coordination of the study.

**SPN **participated in the design of the study and performed the mathematical analysis.

**CEK **conceived of the study and participated in its design and coordination.

**FGS **and **JZ **helped to draft the manuscript.

They all approved the final version of the manuscript.

## Pre-publication history

The pre-publication history for this paper can be accessed here:



## Figures and Tables

**Table 1:** Intake questions asked of participants in GIS

-Age/Male/Female
-Daily occupation? (School/work/home/other)
-Means of transportation? (Car/bike/public)
-How many times per year suffering from cold/ILI? (<2/2–5/>5)
-Recently vaccinated against flu? (Yes/no)
-Do you have asthma or diabetes? (No/asthma/diabetes)
-Do you smoke? (Yes/no)
-Do you follow the recommendations of the Dutch Food and Nutrition Centre? (No/occasionally/daily)
-Do you use vitamins? (No/occasionally/every day)
-Do you follow a diet? (No/vegetarian/veganistic/low-calorie)
-Do you exercise? (<1 hour/week; 1–4 hours; >4 hours per week)
-How many people at home? (Alone/with adults/with adults and children)
-Do the children attend school or nursery? (No/nursery/school)
-Do you have pets? (No/cat(s)/dog(s)/bird(s)/other)

**Table 2:** Questions asked about symptoms of ILI

-No symptoms?	Cough?
-Running nose?	Headache?
-Sore throat?	Chest pain?
-Muscle pain?	Diarrhea?
-Abdominal pain?	Cold shivers?
-Nausea	Irritated eyes?
-Sudden fever? (No/yes/don't know)	
-How high was the fever? (37C/between 37C and 40C, in steps of 0.5 degree)	

Definition of ILI: Fever >38C, that started suddenly, plus headache or muscle pain, plus at least one of the respiratory symptom: running nose/coughing/sore throat/chest pain

**Table 3:** Comparison between distribution of gender and age in GIS and the Dutch population

	**Males (%)**			**Females (%)**		
**Age**	**GIS**	**DP**	**R (CI)**	**GIS**	**DP**	**R (CI)**
**0–10**	2.8	14.6	0.15 (0.13–0.18)	2.8	14.4	0.16 (0.14–0.19)
**11–20**	7.9	12.2	0.60 (0.55–0.65)	10.7	11.6	0.90 (0.83–0.97)
**21–30**	11.1	12.8	0.88 (0.81–0.95)	14.4	11.8	1.22 (1.15–1.31)
**31–40**	17.4	16.7	1.09 (1.02–1.16)	19.9	15.4	1.36 (1.29–1.45)
**41–50**	22.2	14.5	1.54 (1.45–1.64)	22.8	13.8	1.69 (1.59–1.78)
**51–60**	22.2	12.7	1.77 (1.67–1.88)	17.2	12.2	1.35 (1.27–1.44)
**61–70**	11.1	8.9	1.27 (1.17–1.37)	9.9	9.0	1.12 (1.04–1.21)
**71–80**	4.7	5.5	0.85 (0.76–0.95)	2.1	7.1	0.29 (0.24–0.33)
**>80**	0.6	2.1	0.29 (0.21–0.39)	0.2	4.7	0.04 (0.02–0.06)

**GIS**: Great Influenza Survey 2003–2004 (Males: n = 6,250; Females: n = 7,050)

**DP**: Dutch population (data from Dutch Statistics; Males: n = 8,065,000; Females: n= 8,234,000)

**R**: Ratio

**CI**: 95% confidence interval (Calculated according to Gardner and Altman (ref. 28)

**Table 4:** Comparison between GIS and LINH population with regard to the prevalence of asthma and diabetes, and influenza vaccination rates

	**LINH**	**GIS**
Prevalence asthma	6.4%	6.9%
Prevalence diabetes	3.5%	2.4% *
Vaccination rate pts. with asthma	73%	68%
Vaccination rate pts. with diabetes	85%	85%
Vaccination rate total population	18%	16%
Vaccination rate 65+	87%	85%

**LINH**: National Information Network of GPs (number of patients: 255,000)

**GIS**: Great Influenza Survey (number of participants: 13,300)

**65+**: persons older than 65 years

* P < 0.005
